# Evaluation of reaction gap-filling accuracy by randomization

**DOI:** 10.1186/s12859-018-2050-4

**Published:** 2018-02-14

**Authors:** Mario Latendresse, Peter D. Karp

**Affiliations:** SRI International/Artificial Intelligence Center, 333 Ravenswood Ave, Menlo Park, 94025 USA

**Keywords:** Flux balance analysis, FBA, Gap-filling, Evaluation, Randomization

## Abstract

**Background:**

Completion of genome-scale flux-balance models using computational reaction gap-filling is a widely used approach, but its accuracy is not well known.

**Results:**

We report on computational experiments of reaction gap filling in which we generated degraded versions of the EcoCyc-20.0-GEM model by randomly removing flux-carrying reactions from a growing model. We gap-filled the degraded models and compared the resulting gap-filled models with the original model. Gap-filling was performed by the Pathway Tools MetaFlux software using its General Development Mode (GenDev) and its Fast Development Mode (FastDev). We explored 12 GenDev variants including two linear solvers (SCIP and CPLEX) for solving the Mixed Integer Linear Programming (MILP) problems for gap filling; three different sets of linear constraints were applied; and two MILP methods were implemented. We compared these 13 variants according to accuracy, speed, and amount of information returned to the user.

**Conclusions:**

We observed large variation among the performance of the 13 gap-filling variants. Although no variant was best in all dimensions, we found one variant that was fast, accurate, and returned more information to the user. Some gap-filling variants were inaccurate, producing solutions that were non-minimum or invalid (did not enable model growth). The best GenDev variant showed a best average precision of 87% and a best average recall of 61%. FastDev showed an average precision of 71% and an average recall of 59%. Thus, using the most accurate variant, approximately 13% of the gap-filled reactions were incorrect (were not the reactions removed from the model), and 39% of gap-filled reactions were not found, suggesting that curation is still an important aspect of metabolic-model development.

## Background

The plethora of genomes sequenced in recent years has given rise to a large number of genome-scale metabolic models. Flux Balance Analysis (FBA) [1, 2] is a steady-state constraint-based modeling approach that has seen widespread application in genome-scale metabolic models. FBA models a condition of growth for an organism by defining four sets: (1) the metabolic reactions of the organism; (2) its nutrients; (3) its secretions (i.e., the metabolites that are secreted); and (4) its biomass metabolites (the biosynthetic products of the metabolic network). The model is said to show growth if all the biomass metabolites can be produced from the nutrients via the metabolic-reaction network. A model may not grow for many reasons, but a common one is missing reactions in the reaction network, or “reaction gaps,” that result from incompleteness in the genome annotation from which the reaction network was inferred. For an introduction on FBA, we refer the reader to [3–6].

Reaction gap-filling, applied to a non-growing FBA model given a specific growth condition (i.e., nutrients and secretions), consists of adding reactions to the reaction network to enable growth (i.e., production of all biomass metabolites). Automatic gap-filling uses an algorithm, a “gap-filler,” to find these reactions to add. But how effective are such gap-fillers? Past publications on reaction gap-filling have performed very limited evaluations, and they have not directly asked how accurately gap-fillers can reconstruct a known metabolic network.

The central idea behind our approach to testing gap-filler accuracy is to randomly remove a set of reactions *Δ* from a metabolic network *R* to produce a modified network *R*^′^. We then run a gap-filler on *R*^′^ and ask: *to what degree do the reactions suggested by the gap-filler match* Δ*?* We call solutions that exactly match *Δideal* solutions because they exactly recover the original network *R* — that is, had a gap-filler encountered *R*^′^ in a real-world run, it would have perfectly reconstructed the biologically correct starting network *R*. For our experiments, *R* is the EcoCyc-20.0-GEM metabolic model for *Escherichia coli* derived from the EcoCyc database. EcoCyc is likely to contain the most accurate genome annotation and metabolic network of any free-living organism because EcoCyc has been curated from 32,000 publications. Starting with an accurate model means we have more confidence in evaluating a gap-filler’s solutions than if we started with an inaccurate model, if all other factors are kept the same.

We also compute the precision and recall metrics on the results obtained. Precision tells us what fraction of the reactions predicted by the algorithm were in the set of reactions removed. Recall tells us what fraction of the reactions removed were recovered by the algorithm.

MetaFlux is the metabolic modeling component of the Pathway Tools software [7, 8]. MetaFlux contains two gap-filling algorithms, both of which run within the Pathway Tools environment only. Because both algorithms aid the user in developing metabolic models, we refer to them as *development modes* of MetaFlux. One gap-filler uses mixed-integer linear programming (MILP) and is called *General Development Mode* (GenDev); the second uses linear programming (LP) and is called Fast Development Mode (FastDev). We implemented 12 variants of GenDev during the course of this work using two linear solvers (SCIP and CPLEX) for solving the Mixed Integer Linear Programming (MILP) problems for gap filling; three different objectives were applied; and two MILP methods were implemented. We found large variation among the performance of these 12 variants in terms of speed, accuracy, and value of the information returned to the user.

Although the gap-filling algorithm is an important determinant of gap-filling performance, the accuracy of a gap-filler will also be highly dependent on both the size and the quality of the reaction database from which it draws. GenDev and FastDev both draw their reactions from the MetaCyc database [9]. MetaCyc contains significantly more reactions than is used by some other gap-fillers (13,924 reactions in MetaCyc version 20.5 from December 2016 versus, for example, the comparable KEGG database with 10,411 reactions in its version 81.0 from January 2017). MetaCyc curators attempt to balance all MetaCyc reactions, although a small number of reactions (249) are unbalanced because they were unbalanced when published and it is unclear how to balance them (our gap fillers automatically ignore unbalanced reactions). MetaCyc curators also annotate reaction direction carefully since correct directionality is important to gap filling [10]. MetaCyc also contains much more extensive curation and explanation than does the KEGG database, including 7800 textbook-equivalent pages of mini-review summaries (compared with a negligible number in KEGG), and 51,000 citations to the literature (compared with a negligible number in KEGG).

We note that although a number of other algorithms are described as performing gap filling, they actually solve a range of different problems from enabling flux of just the biomass metabolites in a model to enabling flux through all metabolites in a model. Although enabling flux through all metabolites in a model is in general a worthy scientific goal, we see it as a problematic goal for evaluation purposes because we do not yet have any gold-standard, highly curated model for which all metabolites are unblocked — even for *E. coli*. Previous gap-filling algorithms are as follows.

ModelSEED [11] uses a technique similar to Technique B, using Big M with CPLEX. They reported several hours of execution time to solve some of the gap-filling problems. To increase gap-filling speed, the current (November 2017) gap-filling algorithm provided by the ModelSEED website uses a modified version of the FastDev technique.

The GapFind/GapFill [12] applications use MILP to find blocked metabolites and gap-fill a reaction network, respectively. GapFill uses KEGG or MetaCyc as a source of candidate reactions to add to the reaction network such that a specific metabolite becomes unblocked.

FastGapFill [13] gap-fills a compartmentalized reaction network. It has been tested using the KEGG database as a source of candidate reactions. Its algorithm is based on LP and greedily expands a set of candidate reactions. The algorithm does not guarantee a minimum set of suggested reactions to add, that is, the result is not necessarily the smallest possible set of reactions to gap-fill the reaction network.

Mirage [14] gap-fills a reaction network by using phylogenetic and expression profiles besides a set of candidate reactions. It uses the KEGG database for the set of candidate reactions. Its algorithm combines randomized selection of reactions and a greedy heuristic to prune this selection.

Ponce de Leo et al. use gap filling to unblock the blocked reactions in many metabolic models [10] using the fastcore algorithm [15].

A recent paper by Prigent [16] is most similar to our work. They also take the approach of randomly degrading an *E. coli* metabolic model (the iJR904 model [17]). Several differences exist between our approaches. Prigent et al. use randomly generated biomass functions rather than the actual biomass function; whether their results will be applicable to real biomass functions is unclear. Further, Prigent et al. use topological gap-filling, which ensures topological reachability of a set of metabolites, but in some cases does not enable flux through the specified biomass metabolites, which from our perspective is the primary purpose of gap-filling. Finally, Prigent et al. do not report correctness statistics for their algorithm or the others that they evaluate, in terms of either precision or recall against the set of reactions that were originally removed.

In the following sections, we present the two MetaFlux gap-fillers, our computational gap-filling experiments, and a discussion of the results.

## Methods

### General development mode

Given a metabolic network, a set of nutrients for growth, and a set of biomass metabolites, the GenDev gap-filler [18] is called when the current set of reactions within the metabolic network cannot produce a positive flux for every biomass metabolite given those nutrients.

We implemented three GenDev techniques, and for each technique we studied four variations of it using CPLEX or SCIP as the solver, and using indicators or Big M. In Technique A, each biomass metabolite participates independently in the objective function and no biomass reaction is involved. Technique A [18] reports in its output the largest number of biomass metabolites that can be synthesized by the metabolic network, either with no reactions added to the network, or with the addition of a minimum-cost set of candidate reactions. Thus, Technique A identifies to the user what biomass metabolites cannot be produced by the model even with the addition of all reactions from MetaCyc, which is important information when developing a model. We call this metabolite set the *non-producible biomass metabolites.*

Technique B determines the minimum-cost set of reactions that must be added to the network to produce *all* the biomass metabolites. In Technique B, the biomass metabolites do not directly participate in the objective function of the MILP formulation, but the biomass reaction, in which all biomass metabolites participate, is constrained to have a flux greater than 10^−3^. This value is constant, that is, it does not depend on the model or any of the parameters specified in the model. This value has been selected such that a model is considered growing if and only if the model can produce a flux greater than this value. Technique B was implemented during this work because, as we will show, it yields more solutions that show growth when the model is solved than does Technique A. This increase in valid solutions is mostly due to constraining the biomass reaction to have a flux of at least 10^−3^. Technique B does not identify the non-producible biomass metabolites to the user.

Finally, Technique C is a combination of Technique A and Technique B, that is, a biomass reaction exists, which is constrained to have at least a flux of 10^−3^ (as in Technique B), and the biomass metabolites participate in the objective function. Technique C does identify the non-producible biomass metabolites to the user.

For the three techniques, the candidate reactions are all the metabolic reactions of MetaCyc version 20.5. In addition to gap-filling reactions, GenDev can also [18] gap-fill the nutrients set and the secretions set.

The minimum-cost set is computed based on weights assigned to the biomass metabolites and the candidate reactions. Four weights are used in the experiment done in this paper and in our previous work [18]:

1. 10,000 is assigned to any biomass metabolite (used only in Technique A)
2. 30 is assigned to candidate reactions in the taxonomic range of the organism being gap-filled (*E. coli*)
3. 40 is assigned to candidate reactions of unknown taxonomic range
4. 50 is assigned to candidate reactions outside the taxonomic range of the organism being gap-filled

In the GenDev MILP implementation, a Boolean variable *a*_*r*_ having only values 0 or 1 is used to decide if a candidate reaction *r* should be added to the model. If the value is 1 (interpreted as “true”), the candidate reaction *r* is added; if not (that is, its value is 0 (interpreted as “false”)), the candidate reaction *r* is not added. Similarly, for Technique A, variables *s*_*b*_, one per biomass metabolite *b* to produce, control whether *b* can be produced or not.

Moreover, in GenDev, each boolean variable *a*_*r*_ controls the flux *v*_*r*_ of each candidate reaction *r*. To do so, two methods are well-known, one called “Big M”, the other using the more direct notation of indicator constraints, which we often simply called the “indicators method” (discussed below). For Big M, two constraints are applied to each candidate reaction:

1 $$\begin{array}{@{}rcl@{}} v_{r} - B_{u} a_{r} &\leq 0 \end{array} $$

2 $$\begin{array}{@{}rcl@{}} B_{l} a_{r} - v_{r} &\leq 0 \end{array} $$

where the Boolean variable *a*_*r*_ has value 0 or 1 to control the activation of candidate reaction *r*; continuous variable *v*_*r*_ is for the flux of candidate reaction *r*; *B*_*l*_ and *B*_*u*_ are the lower and upper bounds, respectively, for the flux of any reaction considered to be active. Notice that when *a*_*r*_ is 1, the variable *v*_*r*_ has a value between *B*_*l*_ and *B*_*u*_; whereas when *a*_*r*_ is 0, *v*_*r*_ has value 0^1^. That is, when *a*_*r*_ is 1, the flux of a candidate reaction is forced to be equal to or greater than *B*_*l*_. In our computational experiments, the values used for *B*_*l*_ and *B*_*u*_ were 10^−5^ and 30,000, respectively (these were the best values we found after some experimentation). The bound *B*_*l*_ ensures that the reactions have a minimum acceptable flux to be considered active, whereas *B*_*u*_ limits the flux of any reaction when it is active to avoid numerical overflow.

The indicators notation accepted by most MILP solvers is a more direct way to express the control of each boolean variable *a*_*r*_ over the flux *v*_*r*_ of each candidate reaction *r*. This notation has the form

3 $$\begin{array}{@{}rcl@{}} a_{r} = 0 &\to v_{r} = 0 \end{array} $$

4 $$\begin{array}{@{}rcl@{}} a_{r} = 1 &\to v_{r} \geq B_{l} \end{array} $$

5 $$\begin{array}{@{}rcl@{}} a_{r} = 1 &\to v_{r} \leq B_{u} \end{array} $$

where *a*_*r*_, *v*_*r*_, *B*_*l*_, and *B*_*u*_ have the same meaning as for the Big M method. The symbol → means “implies”. The indicators are such that when *a*_*r*_=0 then the flux of reaction *r* is zero, whereas if *a*_*r*_=1 then the flux of *r* is between *B*_*l*_ and *B*_*u*_. As can be seen, no numerical operations, such as multiplication or addition, are involved in these constraints. That approach reduces explicit numerical errors. But more importantly, there is an explicit indication to the solver that variable *a*_*r*_ directly controls the values of variable *v*_*r*_, which is hidden in the Big M method. Solvers will typically treat these indicators in such a way that when a solution is found, these implications are true. On the other hand, as will be seen in the experimental results of “Computational experiments” section, indicators are more computationally expensive compared to the Big M method.

The objective function of the MILP formulation in Technique A and Technique C is

6 $$ \text{max}\, \left(\sum_{b} 10000 s_{b}\right) - \left(\sum_{r} w_{r} a_{r}\right)   $$

where the Boolean variable *s*_*b*_ controls the inclusion of the biomass metabolite *b* and *w*_*r*_ is one of the three reaction weights (i.e., 30, 40, 50). With such a high value of 10,000, the objective is to synthesize as many biomass metabolites as possible by using a minimum weighted set of candidate reactions.

The objective function in Technique B is

7 $$ \text{max}\, - \left(\sum_{r} w_{r} a_{r}\right)   $$

where the variables *w*_*r*_ and *a*_*r*_ are the same as in Technique A. A constraint *v*_biomass_>10^−3^ is also added to the formulation to ensure that the biomass reaction, involving all biomass metabolites, is sufficiently above zero. This constraint is also applied by Technique C, but where the number of biomass metabolites in the biomass reaction is variable (see next paragraph).

In Technique C, the constraints to enforce the FBA steady state for the biomass metabolites are handled differently than the other two Techniques. Recall that an FBA LP (or MILP) formulation constrains each metabolite to be flux balanced to obtain a steady state, that is, the sum of all production fluxes are constrained to be equal to the sum of all consumption fluxes for that metabolite. Such a constraint is also applied to the biomass metabolites. However, Technique C allows a variable number of biomass metabolites to participate in the biomass reaction, that is, once the MILP formulation is solved, the solution may contain a subset of the original list of biomass metabolites provided (such a solution points to the impossibility of producing some biomass metabolites even when considering candidate reactions to add to the reaction network). Therefore, a steady state constraint must be written to allow this **dynamic** behavior of the biomass reaction. Using indicators, such a constraint for a metabolite *M* is formed by two conditional constraints of the form

8 $$\begin{array}{@{}rcl@{}} s_{M} = 1 &\rightarrow c_{b} v_{\text{biomass}} + \sum c_{r} v_{r} = 0 \end{array} $$

9 $$\begin{array}{@{}rcl@{}} s_{M} = 0 &\rightarrow \sum c_{r} v_{r} = 0 \end{array} $$

where *s*_*M*_ is a binary variable controling the inclusion or exclusion of metabolite *M* in the biomass reaction, *c*_*r*_ is the stoichiometric coefficient of *M* in reaction *r* producing or using metabolite *M* (with the appropriate positive or negative sign according to whether *M* is used or produced by *r*) and *c*_*b*_ is the coefficient of *M* in the biomass reaction. As can be seen, when *M* is included in the biomass reaction (i.e., *s*_*M*_=1), the constraint includes the biomass reaction flux, but when *M* is excluded (i.e., *s*_*M*_=0), the biomass reaction flux is not constrained by the production of *M*.

When using Big M, the previous two constraints are instead written using four constraints, two constraints per *s*_*M*_ value (values 0 or 1), that is, we use:

10 $$\begin{array}{@{}rcl@{}} c_{b} v_{\text{biomass}} + \sum c_{r} v_{r} + 10 s_{M} &\leq 10 \end{array} $$

11 $$\begin{array}{@{}rcl@{}} c_{b} v_{\text{biomass}} + \sum c_{r} v_{r} - 10 s_{M} &\geq -10 \end{array} $$

12 $$\begin{array}{@{}rcl@{}} \sum c_{r} v_{r} - 10 s_{M} &\leq 0 \end{array} $$

13 $$\begin{array}{@{}rcl@{}} \sum c_{r} v_{r} + 10 s_{M} &\geq 0 \end{array} $$

The first two constraints implement the case when *s*_*M*_=1, that is, when *s*_*M*_=1 the first two constraints translate to $c_{b} v_{\text {biomass}} + \sum c_{r} v_{r} = 0$ while the last two constraints translate to $-10\leq \sum c_{r} v_{r} \leq 10$. The last two constraints implement the case when *s*_*M*_=0 by a similar reasoning. The value 10 used in this Big M method was experimentaly chosen. If that value is too large, it appears that numerical imprecision of the solver cannot enforce properly the equality to zero.

### Fast development mode

MetaFlux’s FastDev mode uses LP instead of MILP to gap-fill a reaction network. The running time of FastDev is therefore more predictable and, in most cases, faster than GenDev. The running time of FastDev is more predictable, because it always solves a limited number of linear programs; whereas GenDev, using MILP, might try to solve a very large number or even an unlimited number of linear programs. The complete algorithm of FastDev is described in [19], but the following gives a simplified description.

The main objective of FastDev is not finding the minimum-cost set of reactions to enable production of all biomass metabolites; rather, it is maximizing flux through the biomass reaction. In some cases, fulfilling that objective is equivalent to enabling production of all biomass metabolites; but in other cases, FastDev will find solutions that increase biomass flux beyond a solution that GenDev (Technique A) would find.

The objective function of the FastDev LP formulation is

14 $$ \text{max}\, g v_{\text{biomass}} - \left(\sum_{r} w_{r} v_{r}\right)  $$

where *g* is a numerical parameter, integer or not, selected by the FastDev algorithm; *v*_biomass_ is the flux of the biomass; the weights *w*_*r*_ are the same as in GenDev; and *v*_*r*_ is the flux of the candidate reaction *r*. Using binary search, the FastDev algorithm tries several values of *g* between 0 and *n*, where *n* is the number of candidate reactions. That means that ⌈log*n*⌉ values are tried, which is currently 14 (for MetaCyc 20.5, ⌈log15,000⌉=14) and will unlikely be more than 17 for the foreseeable future, because that would require more than ⌊2^17.5^⌋=185,364 reactions in MetaCyc. All the constraints of FastDev have the same form as in the LP formulation, but more such constraints exist in FastDev because the candidate reactions are also in the LP formulation.

The objective function above includes all candidate reactions as in GenDev, but no integer variables are used to turn the reactions on or off; instead, the non-integer variables *v*_*r*_ for the flux of the reaction *r* have a similar role. After this LP formulation is solved, if the flux *v*_*r*_ is set to a non-zero value, the candidate reaction *r* is active and suggested to be added to the model.

Assume that only one set of candidate reactions *R* such that, once added to the model, the biomass flux *v*_biomass_ becomes a non-zero value (i.e., adding the reactions of *R* makes the model grow). We can show that objective function (14) is strictly positive when the variables *v*_*r*_>0 are set to the fluxes of the reactions *R* that shows the model growing, and for a large enough positive value of *g*. The FastDev algorithm does find such a value for *g*. Because this would be the only case where objective function (14) is non-zero (that is, its maximum value), the LP formulation would suggest adding the reactions *R*.

If two sets *R*_1_ and *R*_2_ of candidate reactions make the model grow, and *R*_1_ is a strict subset of *R*_2_, then *R*_1_ would be selected if *g* is smaller than all weights *w*_*r*_. The selection of *R*_1_ is considered better as it provides a smaller set of reactions to add. In general, keeping *g* smaller than the weights *w*_*r*_ ensures that FastDev finds a minimal set of reactions (i.e., a set of reactions from which no reaction can be removed and still obtain growth). A minimal set is not necessarily a set with the smallest number of reactions that can make the model grow (i.e., a minimum set), because another minimal set may have a smaller number of reactions that also does so. On the other hand, keeping *g* smaller than all weights *w*_*r*_ and finding a set of suggested reactions showing growth are not always possible. This is typically the case when setting *g* to a large enough value such that enough candidate reactions become active and setting *v*_biomass_ to a non-zero value are necessary.

In summary, FastDev may find the smallest set of reactions to enable production of all biomass metabolites, but such an outcome is not guaranteed.

### Computational experiments

We have performed the following computational experiments using MetaFlux version 21.5 to study the strengths and weaknesses of GenDev (Techniques A, B and C) and FastDev. The publicly released version of MetaFlux uses Technique A with SCIP and Big M; all other gap-filler variations are not in the public release. We used version 20.0 of the genome-scale metabolic model [20] derived from the EcoCyc database [21] (EcoCyc-20.0-GEM). The model grows aerobically on glucose and produces 87 biomass metabolites. We solved the model (meaning we solved a flux balance analysis formulation for the model) to determine the set of active, non-generic, reactions^2^. When solved, the biomass flux of the model was 1.0030 mmol/gDW/hr, with 476 active reactions (that is, reactions that carry flux). We define the set of *restricted active reactions* as the subset of the active reactions that are not transport reactions and where instantiated reactions are replaced by their generic reactions. There were 400 restricted active reactions.

Each computational experiment consisted of removing, from the reaction network *R* of the model, a set of randomly selected restricted active reactions to create a perturbed reaction network *R*^′^. We then separately used GenDev (Techniques A, B and C) and FastDev to gap-fill the resulting networks *R*^′^. We ran 100 cases for each of six different numbers of removed reactions, (i.e., for 1, 2, 5, 10, 20, and 50 randomly selected active reactions) for a total of 600 cases. Gap-filling was performed separately on those 600 cases by GenDev (Techniques A, B and C) and FastDev. The sets of nutrients, secreted metabolites, and biomass metabolites were held constant for all these cases.

For each of the 600 GenDev cases for each of Techniques A, B and C, we ran both Big M and the indicators method on each of two solvers, SCIP (version 2.0.1) and CPLEX (version 12.6.3.0), for a total of 7200 computational experiments^3^. A maximum of 30 min of elapsed time was allowed for each GenDev run. For CPLEX, it was not possible to control the total amount of CPU time used because CPLEX uses multiple cores, whereas SCIP does not. This difference did favor CPLEX, but it is not the intention of this work to compare these two solvers, but to be able to use the indicators method for which only CPLEX, as we will see, could solve most cases in a reasobale amount of time.

All the 7200 gap-filler experiments for GenDev (Techniques A, B and C) and the 600 experiments of FastDev (a total of 7800 experiments) considered all the metabolic reactions of MetaCyc (version 20.5) as candidate reactions for gap-filling, which includes all the reactions of EcoCyc. Therefore, we expected to find a solution with growth for all 7800 experiments (that is, a set of suggested reactions to add to *R*^′^ to obtain growth), because the randomly deleted active reactions were among the 13,469 metabolic reactions of MetaCyc.

For GenDev and FastDev, we used the default values for the weight parameters. More specifically, for the weights on the candidate reactions to add, the weights 30, 40, and 50 were used for in taxonomic range, for unknown taxonomic range, and for outside taxonomic range reactions, respectively. No reactions from the model or from the candidate reactions were tried in the reverse direction for the non-reversible reactions.

For GenDev using Big M, for Technique A, B and C, each solution was found in less than 120 s by the SCIP solver used by MetaFlux^4^. For the same methods and techniques, CPLEX found each solution in less than 30 s. For FastDev, each solution was found in less than 60 s by the SCIP solver^5^. CPLEX was not used with FastDev.

For GenDev using indicators, for all three Techniques, the SCIP solver could not solve in less than 30 min of computation most of the 600 cases. This fact is noted by “na” in Tables 1, 2 and 3, because we had too few solutions for SCIP using indicators to present meaningful statistics. The CPLEX solver was able to solve almost all cases using the indicators, although the running time was much higher than for the Big M method. For cases where 10 or fewer reactions were removed, CPLEX was able to find a solution in less than one minute, but many cases where 20 or more reactions were removed CPLEX reached the time limit of 30 min, although in almost all cases a solution was returned that was feasible and where all biomass metabolites were produced.

**Table 1:** GenDev Technique A: Results for 2400 computational experiments using the General Development Mode (GenDev) with each biomass metabolite as an independent part of the objective function, that is, no constraint is applied to the overall biomass reaction

(1) # rxns removed	**1**		**2**		**5**		**10**		**20**		**50**	
(2) Rxns suggested are identical to rxns removed	48	na	22	na	3	na	0	na	0	na	0	na
	**52**	50	22	**23**	3	**7**	0	0	0	0	0	0
(3) No rxns suggested	**36**	na	15	na	0	na	0	na	0	na	0	na
	**36**	25	**17**	13	**1**	0	0	0	0	0	0	0
(4) Rxns suggested are a strict subset of rxns removed	0	na	40	na	43	na	27	na	8	na	0	na
	0	0	**42**	35	40	7	21	**36**	9	**23**	0	**4**
(5) # rxns suggested is equal to # rxns re-moved	11	na	9	na	11	na	4	na	0	na	0	na
	9	**18**	8	**20**	13	**51**	**7**	4	**2**	0	0	0
(6) # rxns suggested is less than # rxns re-moved	0	na	8	na	40	na	64	na	91	na	98	na
	0	0	9	5	37	6	63	60	89	77	99	96
(7) # rxns suggested is greater than # rxns removed	5	na	6	na	**3**	na	5	na	1	na	0	na
	**3**	7	**2**	4	6	33	9	**0**	**0**	**0**	**0**	**0**
(8) # cases where no solution was found	0	na	0	na	0	na	0	na	0	na	2	na
	0	0	0	0	0	3	0	0	0	0	1	0
Precision (%)	77	na	81	na	79	na	80	na	79	na	77	na
	**83**	69	**83**	78	75	**84**	79	**85**	79	**89**	76	**89**
Average precision (%)						79	na					
						79	**82**					
Recall (%)	52	na	**51**	na	54	na	52	na	52	na	52	na
	**54**	**54**	48	**51**	50	**59**	**57**	**57**	57	**59**	57	**59**
Average recall (%)						52	na					
						54	**56**					
					193 (32%)		na					
					223 (37%)		144 (24%)					

A GenDev solution is a set of suggested reactions to add to a degraded model to obtain growth. Each cell of this table is a 2x2 matrix whose first row is for the SCIP solver; the second row is for the CPLEX solver; the first column is for the Big M method; the second column is for the indicators method. For example, the cell on the first row and first column, has a matrix with value 48, which corresponds to SCIP using the Big M method, whereas the value 52 is for CPLEX (using the same method) and the value 50 is for CPLEX using indicators. A result “na” (not available) applies to SCIP using indicators — in most cases that solver could not find a solution in less than 30 min of computation. Each column of the table represents 400 computational experiments based on randomly removing the same number of active reactions from a base model (in each cell of the table, 100 experiments were run for each of the four cells in the matrix). The first row “# rxns Removed” lists the number of active reactions randomly removed. The other rows divide the 100 cases in each column into solutions of different types; for each cell of the small matrices, rows 2-8 of every column sum to 100. The best numbers are in bold, which could be the maximum or the minimum value depending on the row

**Table 2:** GenDev Technique B: Results for 2400 computational experiments when using the General Development Mode (GenDev) with the biomass reaction constrained to a minimum flux of 10^−3^ but **not** as part of the objective function

(1) # rxns removed	**1**		**2**		**5**		**10**		**20**		**50**	
(2) Rxns suggested are identical to rxns removed	58	na	29	na	4	na	0	na	0	na	0	na
	59	**65**	26	**32**	4	**8**	0	**1**	0	0	0	0
(3) No rxns suggested	25	na	12	na	0	na	0	na	0	na	0	na
	25	25	12	12	0	1	0	0	0	0	0	0
(4) Rxns suggested are a strict subset of rxns removed	0	na	35	na	37	na	21	na	8	na	0	na
	0	0	32	**37**	38	**53**	20	**49**	7	**20**	0	**1**
(5) # rxns suggested is equal to # rxns re-moved	**10**	na	9	na	16	na	8	na	**3**	na	0	na
	9	9	**13**	11	**17**	9	**10**	3	1	0	0	0
(6) # rxns suggested is less than # rxns re-moved	0	na	**8**	na	**29**	na	58	na	83	na	97	na
	0	0	**8**	**8**	**29**	28	**62**	47	**90**	80	**100**	99
(7) # rxns suggested is greater than # rxns removed	7	na	7	na	14	na	13	na	6	na	2	na
	7	**1**	7	**0**	12	**1**	8	**0**	2	**0**	0	**0**
(8) # cases where no solution was found	0	na	0	na	0	na	0	na	0	na	1	na
	0	0	0	0	0	0	0	0	0	0	**0**	**0**
Precision (%)	81	na	80	na	75	na	78	na	80	na	77	na
	83	**87**	76	**84**	77	**86**	78	**90**	78	**87**	77	**88**
Average precision (%)						78	na					
						78	**87**					
Recall (%)	64	na	**55**	na	58	na	58	na	**60**	na	59	na
	65	**66**	54	**55**	60	**61**	59	**62**	58	**60**	59	**60**
Average recall (%)						59	na					
						59	**61**					
# Solutions that do not show growth					0		na					
					0		4 (0.6%)					

The meaning of rows and columns are the same as in Table 1

**Table 3:** GenDev Technique C: Results for 2400 computational experiments when using the General Development Mode (GenDev) with a **dynamic** biomass reaction constrained to a minimum flux of 10^−3^ but **not** as part of the objective function

(1) # rxns removed	**1**		**2**		**5**		**10**		**20**		**50**	
(2) Rxns suggested are identical to rxns removed	**63**	na	**28**	na	6	na	0	na	0	na	0	na
	59	54	24	27	5	**7**	0	**1**	0	0	0	0
(3) No rxns suggested	25	na	12	na	0	na	0	na	0	na	0	na
	25	25	12	12	0	0	0	0	0	0	0	0
(4) Rxns suggested are a strict subset of rxns removed	0	na	**34**	na	38	na	23	na	7	na	0	na
	0	0	**34**	33	35	**49**	19	**37**	8	**20**	0	0
(5) # rxns suggested is equal to # rxns re-moved	7	na	11	na	15	na	6	na	**5**	na	2	na
	**9**	**9**	**14**	13	**19**	10	**10**	3	3	0	0	0
(6) # rxns suggested is less than # rxns re-moved	0	na	7	na	25	na	59	na	82	na	97	na
	0	0	6	**9**	29	**32**	**64**	59	**88**	80	99	**100**
(7) # rxns suggested is greater than # rxns removed	**5**	na	8	na	16	na	12	na	6	na	1	na
	7	12	10	**6**	12	**2**	7	**0**	1	**0**	1	**0**
(8) # cases where no solution was found	0	na	0	na	0	na	0	na	0	na	0	na
	0	0	0	0	0	0	0	0	0	0	0	0
Precision (%)	**87**	na	**80**	na	76	na	78	na	77	na	74	na
	83	75	77	76	76	**82**	78	**87**	78	**87**	75	**88**
Average precision (%)						79	na					
						78	**83**					
Recall (%)	**67**	na	**56**	na	**61**	na	59	na	59	na	59	na
	53	59	53	51	60	60	59	**60**	58	**60**	58	**60**
Average recall (%)						**60**	na					
						57	58					
# Solutions that do not show growth						**0**	na					
						**0**	1					

The meaning of rows and columns are the same as in Table 1

## Results

A statistical summary of the results of applying GenDev Technique A on the 600 cases is presented in Table 1. Each cell of that table has a small 2x2 matrix for the four experiments for each case. The matrix values are described in the table caption. Similarly for Technique B and Technique C, the results are presented in Tables 2 and 3, respectively. For FastDev, the results are presented in Table 4. There are only single values in each cell for FastDev because it was applied using only SCIP, and the Big M and indicators method do not apply to FastDev, which uses LP, not MILP. We did not use CPLEX for FastDev because we chose to focus on the MILP capability of CPLEX, in particular its indicators method. Each table has eight rows labelled 1 to 8, rows for precisions and recalls, and the number of solutions that show no growth once the model was modified according to the suggested reactions to add. The first column gives short descriptions of the meaning of each row, but more explanations follow.

**Table 4:** Results for 600 computational experiments using the Fast Development mode (FastDev)

(1) # rxns removed	**1**	**2**	**5**	**10**	**20**	**50**
(2) Rxns suggested are identical to rxns removed	60	25	4	0	0	0
(3) No rxns suggested	0	0	0	0	0	0
(4) Rxns suggested are a strict subset of rxns removed	0	33	34	17	5	0
(5) # rxns suggested is equal to # rxns re-moved	31	11	16	9	8	0
(6) # rxns suggested is less than # rxns re-moved	0	19	32	59	80	98
(7) # rxns suggested is greater than # rxns removed	9	12	14	15	7	2
(8) # cases where no solution was found	0	0	0	0	0	0
Precision (%)	63	68	75	76	74	72
Average precision (%)	71					
Recall (%)	65	54	59	59	58	58
Average recall (%)	59					
# Solutions that do not show growth	28 (5%)					

The meaning of rows and columns are the same as in Table 1 but using only the SCIP solver without using Big M or indicators because these methods do not apply to FastDev

Row 1 is simply the number of reactions removed for each column. Each column contains 100 cases applied using the two solvers SCIP and CPLEX and the two methods Big M and indicators.

Row 2 shows the number of cases where the set of suggested reactions is the same as the set of removed reactions. For column one, with one reaction removed, this happens about half the time over the 100 cases, whichever solver or method is used, but with Technique B slightly higher than Technique A. Technique B is better on this account.

In Table 1, the number of ideal solutions (row 2, suggesting to add the same set of reactions as those removed) degrades (e.g., for SCIP using no-indicators, 48, 22, 3, 0, 0, 0) when the number of reactions removed increases (i.e., 1, 2, 5, 10, 20, 50). In parallel, the number of solutions suggesting a smaller set of reactions to add (row 6), increases (i.e., 0, 8, 40, 64, 91, 98). This result is simple to explain, because, assuming that the reactions have the same weight, GenDev tends to find the smallest set of reactions that enables growth and such smaller sets, which are different than the reactions removed, are more likely to exist as more reactions are removed. We study two such cases of smaller sets in “Solutions with smaller sets of reactions suggested to be added than the reactions removed” section.

Row 3 shows the number of cases where no reactions are suggested to be added to the model. The number of such cases rapidly decreases as the number of reactions removed increases, which is normal because more essential reactions are removed as more reactions are removed. Examples of such cases are discussed in “Solutions with no reactions suggested” section.

Row 4 presents the frequency at which the sets of suggested reactions are strictly contained in the sets of reactions removed. These numbers do not count the cases where no reactions were suggested (i.e., the empty sets), because these cases are given in row 3. The numbers are high when removing two and five reactions but quickly fall to zero when 20 or more reactions are removed. This quick fall occurs because, in many cases, only one or two suggested reactions are outside the set of removed reactions.

Row 5 shows the number of cases where the number of reactions suggested is the same as the numbers of reactions removed, but the actual reactions differ. This difference is acceptable because other reactions than the one removed can enable growth of the model. GenDev computes only one optimal solution, but several optimal solutions may exist.

Row 7 shows the number of cases where the number of reactions suggested is larger than the number of reactions removed. We would expect that each set of suggested reactions has at most the number of removed reactions, because GenDev searches for a minimum cost set of reactions to add to obtain growth, and because the reactions removed exist in the set of candidate reactions. For many cases, though, the number of reactions suggested to be added to obtain growth is larger than the number of removed reactions.

Row 8 shows the number of cases where the solver could not find any solution. It rarely happens. We explain in “Solutions with larger sets of reactions suggested to be added than the reactions removed” section that imprecision of floating point numbers is likely the cause of these issues.

The rows on precisions and recalls give an overall evalution of the results.

Finally, the last rows of the tables give the number of cases where model growth was in fact not obtained once the suggested solutions were applied to the model. For Technique A the results are very high suggesting that this Technique has a major issue. For Technique B the results are close to zero and for Technique C, for only one case was no growth obtained, due to the time limit reached by the CPLEX solver using indicators.

For FastDev, the results are presented in Table 4. The number of solutions that exactly match the set of reactions removed has the same behavior as GenDev (that is, it decreases as the number of reactions removed increases (e.g., 60, 25, 4, 0, 0, 0) (row 2)). FastDev always suggests adding at least one reaction, no matter the number of reactions removed (row 3 is all zeros). This behavior is expected, because FastDev tries to increase biomass flux and may suggest adding a reaction to reach that goal, as discussed in the first three paragraphs of “Fast development mode” section. That behavior is also reflected by the number of solutions suggesting adding more reactions than the number of reactions removed, as given by row 7 of the table.

## Discussion

What properties does the *E. coli* metabolic network have that make it an appropriate subject for this study? The network is above average in size for a bacterium: EcoCyc 20.0 contains 1637 reactions versus the average network size of 1065 reactions across the 11,000 organisms in BioCyc; EcoCyc contains 4506 genes versus the average genome size of 3488 genes across all of BioCyc. The *E. coli* network has been very highly studied, and has been the subject of multiple modeling efforts [17, 20, 22, 23]. The set of biomass metabolites for the EcoCyc model is large (71 metabolites) compared to most models, meaning that a large subset of the reactions carries non-zero flux. In addition, the MetaCyc DB used as the reference for gap filling contains all of the reactions of EcoCyc, therefore we know it will be able to gap-fill the EcoCyc network when reactions are removed.

### Examination of gap-filling results for GenDev

#### Solutions with larger sets of reactions suggested to be added than the reactions removed

GenDev should theoretically always find solutions with a number of reactions suggested to be added that is not greater than the number of reactions removed. In practice this is not the case as row seven of Tables 1, 2 and 3 shows. The only reason we can find for the non-minimal solutions is numerical imprecision. For example, we think that some of the reactions are made active with a very low flux to enforce some of the constraints although if an exact (integer arithmetic) solver were used, these reactions would not need to be active. A more thorough discussion of the impact of numerical imprecision of non-exact solvers, in the context of FBA, is presented in [24].

#### Solutions with no reactions suggested

When a small number of reactions were removed, GenDev would propose some solutions where no reactions were suggested to be added. These null solutions mean that even when some active reactions are removed, the model still grows. This is not necessarily an error, because some reactions are not essential as the fluxes of other reactions of the model can compensate for the removed reactions. For example, when the reaction RXN0-5461 was removed, no reaction was suggested to be added, and indeed this reaction was inessential. According to Table 1, using SCIP with Big M, this scenario occurred 36 times when one reaction was removed, 15 times when two reactions were removed, while no such cases exist for five or more reactions removed, for a total of 51 such cases.

Some of these cases are possibly in error (that is, once the reactions are removed from the model, the model does not grow when solved). Note that solving a model from which some reactions were removed is different than gap-filling the same model with the same reactions removed because the former used LP, whereas the latter used MILP. These errors occurred for Technique A, but not for Techniques B and C.

#### Solutions with smaller sets of reactions suggested to be added than the reactions removed

Table 1, for SCIP using Big M, shows a total of 301 cases (rows 2, 3, 4, 6) with solutions suggesting a smaller number of reactions than the number of reactions removed, without being strict subsets of the reactions removed. As in the previous section on solutions with no reactions suggested to be added, these 301 cases may contain solutions that are invalid (that is, if we remove the reactions from the model and add the reactions suggested, the model might not show growth).

Similarly as in the previous section, the correct solutions rerouted the fluxes of removed reactions to possibly other reactions, but this time through the suggested reactions to be added, to obtain growth. These errors occurred for Technique A, but not for Techniques B and C.

### Overall performance of the three gap-filling techniques

Tables 1, 2 and 4 show that for both gap-fillers, when as few as 10 reactions are removed, the gap-filler does not find in almost all cases any solutions (across 100 attempts) that exactly match the removed set of reactions. Therefore, we need to treat gap-filler solutions with significant skepticism, and the more reactions that are gap-filled, the lower the probability that the solution will be correct.

On the other hand, how serious is the gap-filler’s solution being not exactly the same as the set of reactions removed? MetaFlux gap-filling is based on the nearly 14,000 candidate reactions present in MetaCyc version 20.5. In general, MetaFlux solutions are not unique — multiple subsets of reactions with the same cost may exist. The number of alternative solutions tends to increase as the number of reactions needed to enable model growth increases. For example, if there is a solution with 10 reactions to gap-fill the network, and each reaction has two equivalent reactions that can substitute for it, the number of solutions would be 3^10^, which is more than 59,000 solutions, a very large number. On the other hand, the found solution likely overlaps to some degree with the reactions within the “correct solution.”

To estimate these overlaps, the recall and precision of the results were calculated as shown in Tables 1, 2 and 4. The precision is the number of reactions removed that were suggested back by the gap filler, divided by the number of reactions suggested. The recall is the number of reactions removed that were suggested back by the gap filler, divided by the number of reactions removed.

The best average precisions are obtained by CPLEX using indicators either for Technique A (82%), Technique B (87%) or Technique C (83%). FastDev has an average precision of 71%. This means that for all four techniques, on average, at least 71% of the reactions suggested were in the correct solution. Conversely, this result means that about one quarter of the reactions suggested were incorrect. We were surprised to see that for Techniques A and B precision (and recall) were essentially constant no matter how many reactions were initially removed from the model.

The best average recalls are obtained by CPLEX using indicators for GenDev Technique A (56%), and B (61%), but for Technique C, SCIP using Big M has the best average recall (60%). FastDev has an average recall of 59%. That means that more than half of the correct reactions were recovered by the four gap-fillers. These percentages would have been higher if the sets of reactions removed had been essential for growth, rather than being active reactions. However, we feel these results are representative because presumably the genome-annotation errors that cause reaction gaps do not preferentially target essential reactions.

Tables 1, 2, and 4 also show that gap-filler solutions are much more commonly smaller than the true set of reactions removed (row 6) than larger than the correct reaction set (row 7). Many gap-fillers intentionally seek a minimum set of reactions and will, therefore, fail to include some correct reactions in their solutions. That is, the cell does not always use a minimum set of reactions.

The most important aspect that differentiates the applicability of Technique A and Technique B is the number of solutions that do not show growth (see the last row of 1). That is, when tested for growth using flux-balance analysis, solutions proposed by the gap filler are not true solutions because they do not enable cell growth. For Technique A, the number of solutions found without growth is experimentally at least 24%. For Technique B, it is less than 1% and Technique C has only one case whith no growth. This result puts Technique A at a strong disadvantage. Technique C combines the best of Techniques A and B to resolve this issue.

In summary, the solutions computed by Technique A show too many errors (no growth) for Technique A to be a viable approach; Technique B has a large precision using indicators but lacks the flexibility of Technique A; and Technique C using indicators provides a good average precision, avoids errors (shows growth in almost all cases) and has the flexibility of Technique A.

### GenDev vs FastDev

Considering the computational experiences described here, what are the different strengths and weaknesses of GenDev and FastDev? One general difference between the two approaches is their objectives. For GenDev, if no numerical errors occurred, it outputs a minimum-cost set of reactions to add to obtain growth, but no such guarantee exists for FastDev because its method is based on a heuristic. GenDev uses MILP, which in general is an NP-hard optimization problem, whereas FastDev uses LP, which is solvable in polynomial time. These are two very different computational complexity classes, where GenDev could take a large amount of time (e.g., hours) to find its answer compared to FastDev (e.g., one minute). The computational time, though, also depends on the methods used, that is, either Big M or indicators. If the Big M method is used the computational time of GenDev and FastDev are similar.

As given in Tables 1, 2, 3 and 4, the recall metrics between GenDev and FastDev are not far apart when using Big M, and GenDev Technique B is closer to FastDev than is Technique A. But for precision, GenDev is superior to FastDev. The difference in precision is understandable, because FastDev tends to propose larger sets of reactions that increase biomass flux. Overall, Technique B using indicators with CPLEX is superior to FastDev and Technique A, but as already mentioned, indicators tend to use substantialy more computational time than FastDev on average.

## Conclusions

We performed an empirical study of the two gap-filling tools — called GenDev and FastDev — within MetaFlux, the metabolic-modeling component of the Pathway Tools software. We compared FastDev and 12 different variations of GenDev based on three techniques, A, B and C; using two solvers, SCIP and CPLEX; and using two methods for MILP formulation, called Big M and indicators. We randomly removed reactions from the EcoCyc-20.0-GEM metabolic model, ran each of the 13 gap-filler variations on the resulting reaction network, and assessed how closely the resulting set of reactions matched the removed set of reactions, which we take to be biologically correct given the large amount of curation and experimental knowledge behind EcoCyc.

Technique A was shown to have a major accuracy issue, because a large percentage (over 24%) of solutions found, using either solvers or methods, did not show growth when the solutions were tested on the model using FBA. However, Technique A provides high flexibility because it identifies to the user the non-producible biomass metabolites.

Technique B was shown to be very accurate — its solutions show growth 99% of the time. On the other, hand, this technique assumes that all the metabolites can be produced with some candidate reactions, which is not always the case when trying to gap-fill a model. Technique A can find a solution even when not all biomass metabolites can be produced.

Technique C produces almost no errors, and has the flexibility of Technique A to identify to the user the non-producible biomass metabolites. However, its accuracy is less than that of Technique B. We intend to use Technique C, most likely with Big M, in the next MetaFlux release in 2018.

Using indicators resulted in better average accuracy when using the CPLEX solver, but the computational cost increased substantially compared to the Big M method. This cost might not always be worth the time to wait for a solution, which makes the Big M method viable. On a solver such as SCIP, the computational cost of indicators becomes prohibitive and is not practically usable.

In general, metabolic models that have been purely automatically gap-filled with no subsequent curation should be treated with caution because of the precisions and recalls found in our study. The best MILP-based GenDev gap-filler using Technique B (CPLEX using indicators) had an average precision of 87% (82% for Technique A), and the LP-based FastDev gap-filler had an average precision of 71%. The best average recall was 61% for GenDev Technique B (CPLEX using indicators) (56% for Technique A) versus 59% for FastDev, meaning that both gap-fillers recovered more than half of the correct reactions.

Many gap-fillers are based on a minimality criterion inspired by the Occam’s razor principle: “More things should not be used than are necessary.” Yet we found that this minimality criterion is sometimes too strong because the GenDev minimality-based gap-filler sometimes recovers only a subset of the reactions deleted in our experiments. That subset is the subset necessary to enable growth of the model, not the set necessary to restore all the deleted reactions. Similarly, real-world gap-filling probably restores only a fraction of the reactions omitted by incomplete genome annotations.

## Abbreviations

FastDev: Fast development mode
FBA: Flux balance analysis
GenDev: General development mode
LP: Linear programming
MILP: Mixed-integer linear programming

## Footnotes

1 Negative flux cannot occur because any reversible reaction was converted to two irreversible reactions and all reactions are bounded by 0.

2 A non-generic reaction applies to specific compounds only (that is, no class compounds are involved in such a reaction). A generic reaction has at least one class compound as a reactant or product. All generic reactions are *instantiated* before the model is solved. Instantiating a generic reaction generally adds one or more reactions to the model, that is, one reaction for each compound that exists in the database as an instance of the compound class of the generic reaction.

3 To be more precise, 7200 computational experiments were attempted, but for SCIP we have not reported the detailed results using indicators because the large majority of them timed out.

4 This time does not include the time for MetaFlux to generate the LP file, which is less than 30 s.

5 In FastDev, the SCIP solver was called 10 times to find each solution, which took an overall of less than 180 s for each solution.
